# Periodate-Mediated Cross-Linking for the Preparation of Catechol Conjugated Albumin Nanoparticles Used for in Vitro Drug Delivery

**DOI:** 10.1021/acsabm.4c01737

**Published:** 2025-02-14

**Authors:** Eda Argitekin, Ozlem Erez, Gulcin Cakan-Akdogan, Yasar Akdogan

**Affiliations:** † Materials Science and Engineering Department, Izmir Institute of Technology, Izmir 35430, Turkey; ‡ Izmir Biomedicine and Genome Center, 35340 Izmir, Turkey; § Izmir International Biomedicine and Genome Institute, Dokuz Eylul University, 35430 Izmir, Turkey

**Keywords:** Catechol conjugation, albumin nanoparticles, catechol cross-linking, periodate, drug carrier

## Abstract

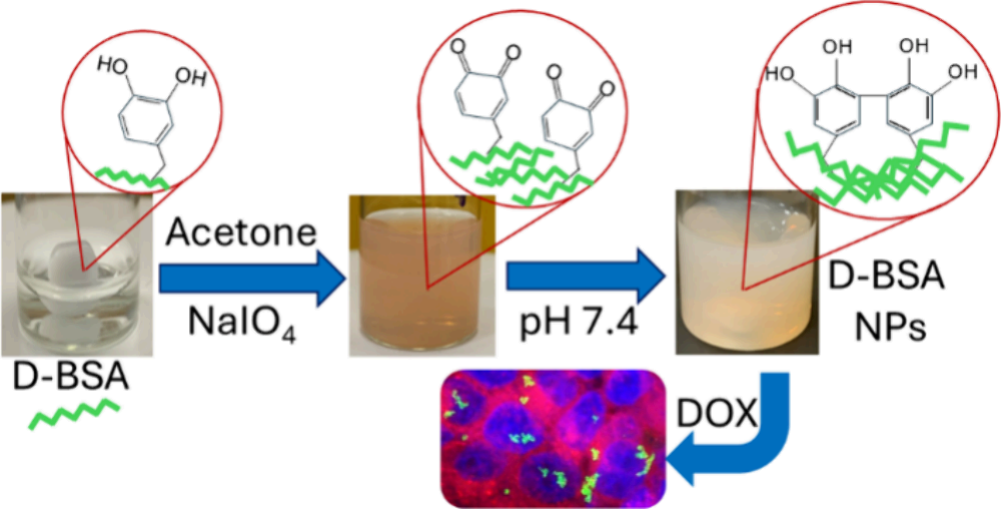

Conjugation of serum albumin protein with catechol-containing dopamine molecules provides an alternative method for the preparation of albumin nanoparticles (NPs). A commonly used desolvation method utilizes glutaraldehyde as a cross-linking agent. Here, the catechol cross-linking mechanism is used instead of glutaraldehyde providing advantages to prevent toxicity and an undesirable reaction of glutaraldehyde with cargo molecules. Covalent cross-linking between dopamine conjugated bovine serum albumin (D-BSA) proteins was obtained in the presence of sodium periodate (NaIO_4_) as an oxidizer. As a result, spherical D-BSA NPs with a uniform size distribution of around 100 nm in diameter and negative zeta potential around −28 mV were prepared. Optimal conditions were reached when a dopamine:IO_4_^–^ molar ratio of 2:1, pH 7.4 of the medium, and acetone as the desolvating agent were used. Furthermore, the obtained NPs display antioxidant properties, have rapid biodegradability in the presence of trypsin, and have a high doxorubicin (DOX) loading (9.1%) with a sustainable drug release. DOX loaded D-BSA NPs also caused up to 90% breast cancer cell (MCF-7) death within 24 h. These results show that drug carrying albumin NPs can alternatively be prepared via covalently cross-linked catechol groups and used in drug delivery studies.

## Introduction

Serum albumin protein which is the most abundant protein in blood plasma has a high transportation capacity for both water-soluble and insoluble drugs.^1^ Serum albumin’s rich binding ability as well as its biodegradability, nontoxicity, and nonimmunogenicity makes it a good candidate for the preparation of nanosized carriers for drugs.^2^ Moreover, serum albumin’s specific receptors called 60 kDa glycoprotein and secreted protein acidic and rich cysteine (SPARCS) which are overexpressed by tumors, such as breast cancer, liver cancer, glioma, prostate cancer, and glioblastoma, facilitate the uptake of albumins by tumor cells.^3−8^

For all these reasons, drug loaded albumin nanoparticles (NPs) are frequently preferred in the field of controlled drug delivery studies and in clinical studies.^9−14^ Abraxane, an albumin-based NP formulation of paclitaxel, is the first human serum albumin (HSA) based drug approved by the FDA in 2005 for the treatment of breast cancer, and it has been also used in the treatment of nonsmall lung cancer, pancreatic cancer, and gastric cancer.^14−16^

Bovine serum albumin (BSA) is often used as a model protein instead of HSA due to its higher abundance and lower cost. The primary structure of BSA is 76% similar to HSA, and the ligand binding properties of both proteins are similar.^17−19^ There are various methods to prepare serum albumin NPs, but the most common and applicable method is the desolvation method, specifically involving glutaraldehyde-based cross-linking.^10,20−22^ Water miscible organic solvents e.g. methanol, ethanol, propanol, acetone, or acetonitrile have been used to desolvate (dehydrate) and denature the protein in aqueous media to obtain protein aggregates.^20,23,24^ Then, cross-linking is necessary for the preparation of stable and uniformly dispersed NPs. Although glutaraldehyde is the most widely used cross-linker, it has toxic properties.^25,26^ This is primarily related to its ability to form covalent bonds with proteins, nucleic acids, and lipids.^27,28^ Undesired cross-linking of biological macromolecules through reaction of glutaraldehyde with functional groups e.g. amine, thiol, phenol, or imidazole leads to cellular toxicity.^29^ For example, the cell survival rate of human TK6 lymphoblasts decreased to 10% after exposure to 20 μM glutaraldehyde for 2 h. This suggests that even low concentrations of glutaraldehyde can cause significant DNA–protein cross-linking.^30^ During the preparation of albumin NPs, glutaraldehyde is consumed for the NP cross-linking mechanism, but the residual amount of glutaraldehyde left could be toxic to cells cultured with NPs. Therefore, the obtained NPs need to be washed several times; after that, they generally do not show significant toxicity for both in vivo and in vitro studies.^9,13,24,31^ Yet, glutaraldehyde still needs to be used with great caution due to its cytotoxic, genotoxic, and environmental toxic potentials.^32^ Exposure to glutaraldehyde can cause irritation and corrosion to the skin, respiratory tract, and eyes.^33^ In addition to cross-linking of proteins, undesirable interactions may also occur between glutaraldehyde and cargo molecules such as nucleic acids or drugs.

Therefore, alternative methods have been developed to obtain serum albumin NPs using different cross-linkers e.g. citric acid, tannic acid, glucose, UV light, *N*-(3-(dimethylamino)propyl)-*N*′-ethylcarbodiimide hydrochloride (EDC), *N*-succinimidyl 3-maleimidopropionate, and metal-catechol bonding.^34−39^ The use of different cross-linkers had an impact on the size, polydispersity index, zeta potential, stability, yield, and cellular toxicity of albumin NPs. For example, while the smaller sizes of BSA NPs were obtained by cross-linking with glutaraldehyde (128 nm), glucose (128 nm), or citric acid (195 nm), larger particles were obtained using ascorbic acid (1692 nm).^34^ The type of cross-linker used in preparation of BSA NPs was shown to affect the viability of human amniotic epithelial cells treated with the NPs. When glucose, UV light, glutaraldehyde, or glucose + UV light was used as a cross-linker, the viability of NP-treated cells decreased to 14%, 16%, 40%, and 76%, respectively.^36^ EDC cross-linked BSA NPs were also synthesized via the formation of peptide bonds between the carboxyl and amine groups of amino acids in the albumins.^35^ The use of EDC decreases the time of NP formation to 3 h in contrast to the overnight incubation time required when glutaraldehyde is used. However, there is a risk of chemical reactions between the EDC and cargo molecules. Previously, we reported use of pH sensitive metal-catechol bonding for albumin cross-linking, as an alternative method.^38^ Dopamine conjugated BSA proteins were cross-linked to prepare pH sensitive NPs in the presence of the V(III) ion. Similarly, catechol conjugated human serum albumin (HSA) proteins were cross-linked via the Fe(III) ion to prepare both NPs and hydrogels.^40^ The pH sensitive nature of catechol-metal coordination provides a compact NP structure due to tris-coordination at a pH above physiological conditions. On the other hand, at the acidic pH, it transforms into mono- and/or bis-coordination, resulting in a more open structure and faster drug release.^38,40^

Here, differently, we prepared BSA NPs via covalently cross-linked dopamines in the presence of an oxidant. Free dopamines can have neurotoxic effects due to radicals, hydrogen peroxide, semiquinones, and quinones formed because of oxidation.^41^ However, here dopamines upon conjugation with BSA were consumed as the cross-linker between BSA proteins.

First, dopamine functionalized BSA proteins (D-BSA) were prepared and desolvated upon the addition of an organic solvent. To obtain spherical colloidal NPs, dopamine conjugated proteins were cross-linked using periodate ions. Oxidation of catechol groups causes molecular coupling between conjugated dopamines, resulting in the stabilization of albumin NPs. By this method, small albumin NPs of around 100 nm were obtained without using toxic glutaraldehyde. Moreover, the use of catechol provides antioxidant ability to albumin NPs. Doxorubicin (DOX), an anthracycline antibiotic with antitumor activity, was used to investigate the drug delivery properties of the obtained NPs. It is regarded as one of the most effective chemotherapeutics and used in the treatment of various diseases including solid tumors e.g. breast, thyroid, bone, ovarian, lung, and soft-tissue cancers.^42,43^ Cellular uptake of the DOX loaded D-BSA NPs and their effects on cell viability were studied using MCF-7 breast cancer cells.

## Materials and Methods

### Materials

Bovine serum albumin (BSA, MW: 66.5 kDa lyophilized powder, >96%), bovine serum albumin fluorescein isothiocyanate conjugate (FITC), *N*-(3-dimethylaminopropyl)-*N*′-ethylcarbodiimide hydrochloride (EDC), *N*-hydroxysuccinimide (NHS), dopamine hydrochloride, sodium(meta) periodate (NaIO_4_), phosphate-buffered saline (PBS), 4-hydroxy-2,2,6,6-tetramethylpiperidine-1-oxyl (TEMPOL), glutaraldehyde solution (8% (v/v) in H_2_O), sodium hydroxide, acetone, ethanol, and propanol were purchased from Sigma–Aldrich (Germany). Doxorubicin hydrochloride (DOX) was purchased from SelleckChem (U.S.A). Trypsin was purchased from GoldBio (U.S.A.). All chemicals and solvents were analytical grade and used without any purification procedures. The pH of the solutions was adjusted with hydrochloric acid and sodium hydroxide. Hydrogen chloride fuming 37% was purchased from ISOLAB (Germany). High purity argon gas was purchased from Güneş Gas (Turkey) with 99.99% purity.

### Methods

#### Preparation of Dopamine Conjugated BSA (D-BSA) Protein

Dopamine conjugated BSA (D-BSA) protein was prepared as previously described.^38^ Dopamine hydrochloride (72 mg) and BSA (72 mg) were dissolved in 3 mL of PBS (0.02 M) at pH 7.2 and 3 mL of ultrapure water, respectively. They were stirred under argon gas for 15 min at 37 °C. The pH of the mixture was adjusted to 6.0 by intermittent addition of HCl (1 M) using a benchtop pH-meter (Starter 3100, OHAUS, Switzerland). Dopamine polymerization was avoided by working in an argon and acidic environment. Then, 72 mg of EDC and 72 mg of NHS were added into the mixture and stirred at 37 °C for 2 h under argon gas. The reaction was stopped by the addition of a 4 M acetate buffer (4 M, pH 6.0). D-BSA was purified by ultrapure water to remove an excess amount of EDC, NHS, and dopamine hydrochloride through 5 cycles of centrifugation (12,000 rpm (9,700 g), 2 min, High Speed Mini Centrifuge, ISOLAB, Germany) by using a centrifugal filter (Amicon Ultra 0.5 mL, molecular cut off: 50 kDa, Merck Millipore, Germany). The yield of the product was 75 ± 8%.

#### Preparation of D-BSA NPs

A 9.5 mg amount of lyophilized D-BSA was dissolved in 1 mL of pure water, and then the pH was adjusted to 7.4 (Starter 3100 benchtop pH-meter, OHAUS, Switzerland) by the addition of NaOH (0.1 M). Then, 5 mL of acetone:water (4:1) (v/v) was added dropwise to the aqueous solution of D-BSA at a rate of 1 mL/min with a syringe pump (SN-50C6T). Afterward, NaIO_4_ (18 μL, 0.081 M) was added drop by drop at 1200 rpm. The pH of the solution was adjusted to pH 4.0, 7.4, or 9.0 by using 0.5 M HCl or NaOH and stirred overnight. D-BSA NPs were transported to 2 mL Eppendorf tubes to be centrifuged at 12,000 rpm (9,700 g) for 15 min. Pellets of D-BSA NPs were washed with ultrapure water by three cycles of centrifugation. Dopamine polymerization which can be detected with a dark brown color was not observed because the amine side of dopamine was covalently conjugated to BSA. The free amine side in the dopamine is required for intercyclization to initiate polydopamine formation.^44^

### Characterization of D-BSA Protein and D-BSA NPs

Molecular masses of BSA and D-BSA proteins were determined by a Bruker Autoflex-III mass spectrometer (smartbeam) MALDI TOF/TOF system. BSA and D-BSA powders were analyzed by an attenuated total reflectance Fourier transform infrared (ATR-FTIR) spectrometer (Nicolet iS50, Thermo Scientific, U.S.A). Zeta potentials of the BSA and D-BSA proteins were analyzed by a dynamic light scattering (DLS) Nano-ZS instrument (Worcestershire, Malvern UK). UV–vis measurements of D-BSA were performed in water at pH 4.0, 7.0, and 9.0 (LAMBDA 365, PerkinElmer, U.S.A.). After the proteins were dissolved in water, the pH values of the solutions were adjusted by the addition of HCl or NaOH using a benchtop pH meter (OHAUS Starter 3100, Switzerland).

An antioxidant study of free dopamine and D-BSA was performed with a CMS 8400 (Adani, Belarus) benchtop X-band electron paramagnetic resonance (EPR) spectrometer at room temperature. It was carried out with a final concentration of 0.7 mM TEMPOL, 10.5 mM dopamine, or 0.7 mM D-BSA prepared in 0.01 M PBS at pH 4.0, 7.4, and 9.0. The mixtures were measured with an EPR spectrometer for 48 h with predetermined time intervals. An antioxidant study of D-BSA NPs was carried out in 0.01 M PBS at pH 7.4. Final concentrations of TEMPOL and NPs are 0.5 mM in the PBS solution. The mixture of TEMPOL and NPs was measured with an EPR spectrometer for 5 days with predetermined time intervals.

NPs were monitored by scanning electron microscopy (SEM, FEI QUANTA 250 FEG, Netherland) to examine the size and shape of the NPs. Purified NPs were dissolved and diluted 3 times with ultrapure water. 4.5 μL solutions of NPs were dropped onto aluminum foil and dried for 1 day. The dried samples were then coated with gold in a vacuum using an EMITECH K550X instrument for SEM imaging. The accelerating voltages ranged between 5 and 7 kV. The size of NPs was measured using Malvern dynamic light scattering (DLS) at a wavelength of 632 nm. The scattering angle was set at 173°. A Malvern dynamic light scattering (DLS) Nano-ZS instrument (Worcestershire, UK) was used to calculate the zeta potentials of NPs.

### DOX Loading to D-BSA NPs

DOX was loaded to D-BSA NPs by the incubation method. 2.8 mg of D-BSA NPs prepared at pH 7.4 was dissolved in 340 μL of PBS buffer (0.005 M, pH 7.4). A 14 μL portion of DOX-HCl (43 mM) was added drop-by-drop to the NP suspension. DOX added NPs were stirred at 300 rpm in the dark, overnight. The final DOX:D-BSA molar ratio is 15:1. DOX-loaded D-BSA NPs were transported to Eppendorf tubes to be centrifuged at 12,000 rpm (9,700 g) for 15 min. UV–vis spectrum of the supernatant was monitored by using a UV–Vis spectrophotometer (LAMBDA 365, PerkinElmer, U.S.A), and unbound DOX was calculated by the calibration curve. To obtain the calibration curve, DOX-HCl stock solutions in PBS (0.005 M, pH 7.4) were prepared. The curve was linear and passed through the origin (R^2^ = 0.9990, n = 17). Entrapment efficiency and drug loading were determined using formulas 1 and 2, respectively
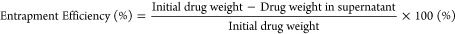
1
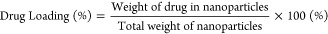
2

### In Vitro Release Studies of DOX from D-BSA NPs

DOX-loaded D-BSA NPs (1.9 mg) were dispersed in 800 μL of a 0.01 M PBS buffer (0.01 M, pH 7.4). The molar ratio of the DOX:D-BSA NP was 15:1. The solution was transferred in 800 μL D-tube dialyzers (MWCO 3.5 kDa, Merck). The dialyzer tube was placed in a beaker containing 32 mL of PBS (0.01 M, pH 7.4) at 37 °C with stirring at 500 rpm. At predetermined time points, 2 mL of a sample was collected and measured by a UV–Vis spectrophotometer to determine the released amount of DOX from the D-BSA NPs. After taking measurements, the 2 mL samples were put back in the beakers.

### Enzymatic Degradation of BSA NPs and D-BSA NPs

Enzymatic degradations of BSA NPs and D-BSA NPs were studied by using trypsin enzyme. 1.0 mg/mL BSA NPs or D-BSA NPs suspended in ultrapure water was mixed with trypsin enzyme. The final concentration of trypsin was adjusted to 50 μg/mL. At 37 °C, the suspensions were stirred at 500 rpm, and the turbidity was obtained by using a UV–Vis spectrophotometer at 565 nm at different time intervals. The absorbance at 565 nm is due to the light scattering effect of the nanoparticles in the solution. Increasing the number of particles in the solution causes greater loss in the intensity of transmitted light, and so the absorbance signal intensity increases with the concentration of NPs. After addition of trypsin, the concentration of the remaining NPs at different times was determined by the calibration curve obtained from different concentrations of D-BSA NPs (Figure S6). Absorption intensities increase linearly versus NPs concentration due to the scattering light effect of nanoparticles in the colloidal solution. The calibration curves were linear and passed through the origin R^2^ = 0.998 and 0.999 for D-BSA NPs and BSA NPs, respectively, for n = 7.

### Cytotoxicity Test

An MTT based cytotoxicity assay was performed after 24 h of incubation with different concentrations of D-BSA NPs, DOX loaded D-BSA NPs, and free DOX with MCF-7 and HEK293T cells. 10,000 cells were seeded in 96-well plates. D-BSA NPs or DOX loaded D-BSA NPs at final concentrations of 0.5, 2.5, 12.5, 25, 50, and 100 μg/mL or free DOX at final concentrations of 0.045, 0.22, 1.12, 2.25, 4.5, and 9.0 μg/mL was added in each well. Absorbance at 570 nm was measured with a BioTek Synergy H1MicroPlate Reader (U.S.A.) for quantification of formazan. The decrease in signals was used for calculating cell survival compared to that of control wells with 100% survival. Each measurement was done in four repetitions, and % viability was calculated.

### Cell Uptake

FITC labeled BSA proteins were used in addition to BSA proteins for cell uptake imaging. FITC labeled BSA was conjugated with dopamines, as explained above. FITC labeled D-BSA and D-BSA proteins were mixed at a molar ratio of 1:4 for NP preparation. MCF-7 cells stained with CM-Dil were seeded at 60,000 cells/well density on 8-well imaging slides. They were treated with 25 μg/mL D-BSA NPs or DOX loaded D-BSA NPs for 4 or 24 h. Excess NPs were removed by washing with 1× PBS-T. Cells were fixed with 4% formaldehyde and poststained with DAPI. Images were acquired with a Zeiss LSM880 (Germany) confocal microscope, with 63X/W objective, and Z-stacks with a 4 μm interval were captured. Only the stacks that correspond to the cells were used to generate maximum Z-projection after background subtraction with ImageJ software.

### Hemolysis Assay

The hemolytic effects of NPs were tested on mouse blood, according to previously published protocols.^38,45^ 3 mL of a mouse blood pool generated from 3 animals was obtained from Izmir Biomedicine and Genome Center (IBG) Vivarium with IBG-Local Ethics Committee approval issued on 30.12.2024. The red blood cells (RBCs) were precipitated from 3 mL of a blood sample (pooled from 3 donors) by centrifugation at 4000 rpm for 15 min. RBCs suspended in PBS were mixed with 4 V of NP dispersions (in PBS) to obtain 0.5, 0.025, and 0.0125 mg/mL final concentrations. The mixture was incubated in a shaker incubator for 4 h at 37 °C and 30 rpm, intact RBCs were removed with centrifugation, and absorbance of the supernatant was recorded at 540 nm.

The percentage of hemolysis was determined by formula 3
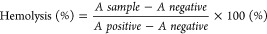
3

## Results and Discussion

### Preparation and Characterization of D-BSA Proteins

Addition of dopamine to the BSA proteins in the presence of EDC yielded dopamine conjugated BSA (D-BSA) proteins. MALDI TOF mass analysis showed that the average number of bound dopamine was 15 when excess dopamine was used (Figure S1(A)). Amide bonds are formed between the amine groups of dopamines and the carboxylic acid groups of amino acids (aspartic acids and glutamic acids).^38^ ATR-FTIR spectroscopy measurements were performed to confirm the formation of new amide bonds after dopamine conjugation (Figure S1(B)). Protein backbone peptide bonds yielded two sharp amide bands at 1650 cm^–1^ and 1533 cm^–1^ and one weak band at 1243 cm^–1^ called amides I, II, and III, respectively.^46^ The number of dopamine conjugated to BSA protein (583 amino acids) is limited, ∼15 dopamines; therefore, the newly formed amide bonds change the spectrum slightly. Still, a detectable signal change at 1243 cm^–1^ was obtained after dopamine conjugation. In addition, the zeta potential of BSA proteins upon dopamine conjugation changed from −16 mV to −7 mV due to the reduced number of free carboxyl groups on the BSA surface (Figure S1(C)).

Catechol groups of dopamines are very reactive and can be easily oxidized at alkaline pH values in an oxygen rich environment or in the presence of oxidizers (periodate, enzymatic oxidants).^47,48^ Yet, they can be used as antioxidants if their protonated forms can be preserved.^49^ Also, serum albumin protein has antioxidant properties, due to the presence of a free thiol group which behaves as a free radical trapper.^50^ In addition, ligands that bind to albumin, including fatty acids, drugs, hormones, and metal ions, can provide direct or indirect antioxidant functions to albumin.^51^ It was previously reported that the D-BSA proteins showed an antioxidant feature due to the catechol groups at pH 7.4, with the EPR method which is not detected for native BSA.^38^

**Figure 1:**
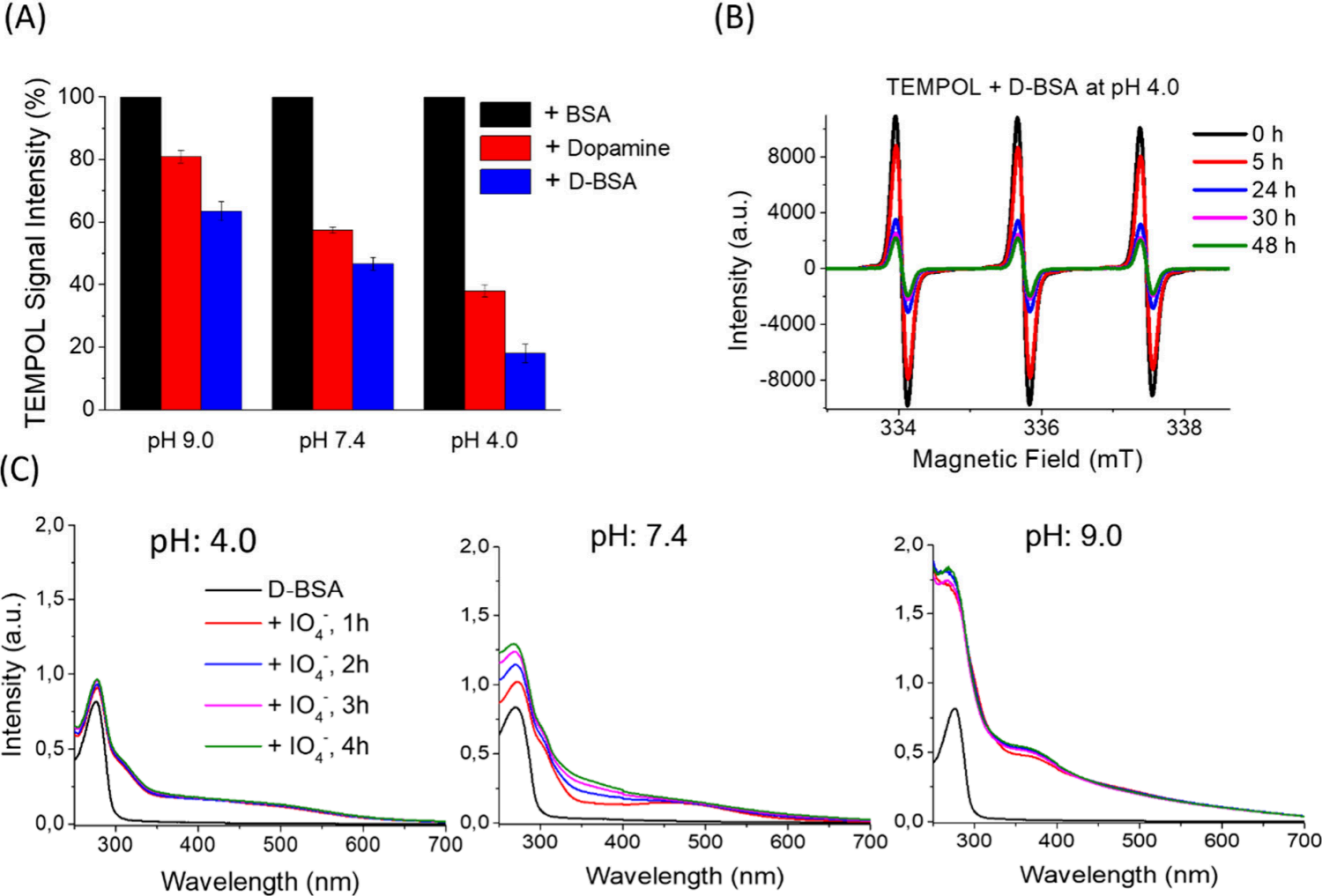
(A) EPR signal intensity change of the TEMPOL radical after 48 h of incubation in 0.01 M PBS buffer upon addition of BSA (black), dopamine (red), and D-BSA (blue) at pH 9.0, 7.4, and 4.0. (B) EPR spectra of TEMPOL before and after addition of D-BSA at 5 h, 24 h, 30, and 48 h in 0.01 M PBS buffer. Final concentrations of TEMPOL, D-BSA, and dopamines are 0.7, 0.7, and 10.5 mM, respectively. (C) UV–vis spectra of D-BSA (0.37 × 10^–5^ M) (black) and time dependent UV–Vis absorption results after addition of NaIO_4_ and adjusting the pH to 4.0, pH 7.4, and pH 9.0. The concentration of IO_4_^–^ is 0.27 × 10^–4^ M.

Here, the antioxidant properties of free dopamine and its conjugated form on albumin were compared using a stable nitroxide free radical of TEMPOL at pH 9.0, 7.4, and 4.0 with EPR spectroscopy (Figure 1(A)). The D-BSA:TEMPOL molar ratio of 1:1 was used. Since each D-BSA contains approximately 15 dopamines, an excessive amount of dopamine was used relative to TEMPOL. The EPR signal intensity of TEMPOL decreased to 80, 57, and 38% in the presence of free dopamines after 48 h at pH 9.0, 7.4, and 4.0, respectively. The antioxidant properties of dopamine were observed mostly at pH 4.0 and at pH 7.4. Oxidation of catechol to quinone at pH 9.0 decreased the antioxidant properties of dopamine. On the other hand, in the presence of D-BSA, containing the same amount of dopamine with the free dopamine experiment, the EPR signal intensity of TEMPOL decreased to 63, 46, and 18% after 48 h, at pH 9.0, 7.4, and 4.0, respectively. More antioxidant properties have been detected, especially at pH 4.0, where dopamines are bound to BSA rather than free dopamines (Figure 1(B)). It suggests that catechol groups of dopamines are protected from oxidation because of the conjugation of dopamines with BSA. A similar phenomenon was reported during adhesive plaque formation in mussels. The oxidation of catechol groups of DOPA amino acids in mussel foot protein (mfp) was found to be reduced if the protein is rich in cysteine content (mfp-6) or hydrophobic residues (mfp-3 slow).^52^

Figure 1(C) shows time-dependent UV–vis spectra of D-BSA at pH 7.4 and after addition of NaIO_4_ followed by the pH adjustment to 4.0, 7.4, and pH 9.0. The conjugated dopamine:IO_4_^–^ ratio required to obtain NPs in the next step was determined as 2:1. The remaining excess dopamines are crucial for the cross-linking steps. Before IO_4_^–^ addition, similar absorption signals originating from BSA protein (due to the aromatic amino acids such as phenylalanine, tyrosine, and tryptophan) and from conjugated dopamine molecules (due to the catechol side) are observed with a maximum at 278 nm at pH 7.4. Upon addition of IO_4_^–^ and then adjusting the pH of the solution to 4.0, the intensity of the absorption peak at 278 nm did not significantly change over time. However, there was a gradual increase at pH 7.4 and an instant increase at pH 9.0 over time, of the absorption peak at 278 nm. This effect is attributed to the formation of phenolic intermediates especially at higher pH values.^53,54^ Moreover, the prominent visible effect observed, especially in the spectrum measured at pH 9.0, could be attributed to the scattering caused by the D-BSA protein aggregates in the presence of IO_4_^–^. The solution became darker and cloudier over time.

Besides, after IO_4_^–^ addition, oxidation of catechol yielded quinone signals around 390–400 nm. Quinone is very reactive; therefore, it transforms quickly, but its signal is obvious at pH 9.0 since alkaline pH helps in the formation of new quinones too. Also, reactions with spare catechol groups and newly formed quinone form a new absorbance signal between 300 and 320 nm, corresponding to α,β-dehydrodopamine. Oxidation of α,β-dehydrodopamine with quinone, molecular oxygen, or residual IO_4_^–^ may initiate formation of dehydro dimers.^53,55^ Also, at pH values 7.4 and 9.0 after IO_4_^–^ addition, the remaining excess catechol groups and newly formed quinones interact to form dicatechol groups, which are detected with signals around 485 nm and an obvious signal increase at 280 nm over time.^53^ The formation of dicatechol groups which is more favored at alkaline pH values can contribute to cross-linking to obtain NPs.^53^ On the other hand, at higher pH values e.g. 9.0, the auto-oxidation of catechols into quinones, which is clearly seen at around 390 nm (Figure 1(C)), can reduce cross-linking density.^53^ Therefore, the pH value of the system is crucial for the cross-linking mechanism and the preparation of NPs.

### Preparation and Characterization of D-BSA NPs Using NaIO_4_

**Figure 2:**
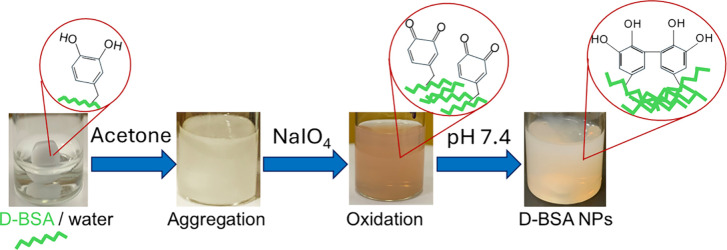
Schematic representation of preparation of D-BSA NPs via covalently cross-linked dopamines in the presence of NaIO_4_ at pH 7.4.

D-BSA NPs were prepared by the desolvation method, followed by the addition of an oxidizing agent of NaIO_4_. The addition of acetone to the aqueous solution of D-BSA proteins (4:1 (v/v), acetone:water) caused turbidity indicating protein aggregation. In addition to the dehydration (desolvation) effect, the denaturation effect of acetone starts protein aggregation (turbidity) in the solution after a 1:1 (v/v) (acetone:water) ratio (Figure 2).^23,24^

**Figure 3:**
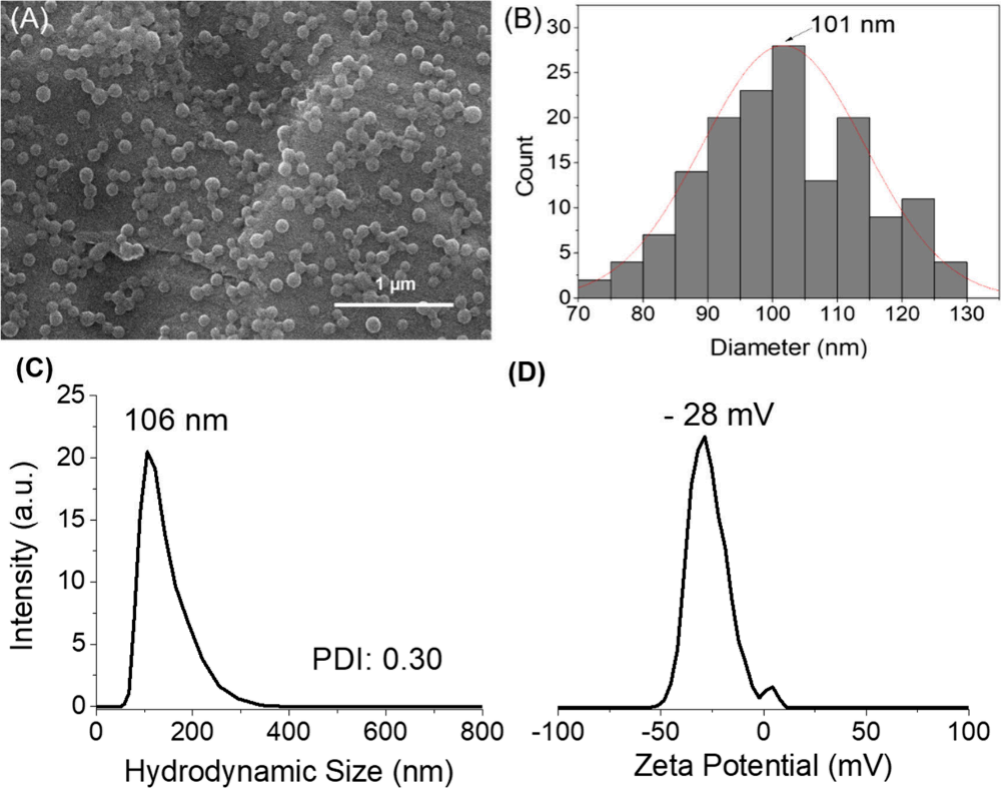
(A) SEM image of D-BSA NPs (pH 7.4) and (B) particle size distribution obtained from the SEM image (A). (C) DLS results of the hydrodynamic size distribution of D-BSA NPs in PBS at pH 7.4. (D) Zeta potential of the D-BSA NPs in water.

After desolvation, protein aggregates were stabilized and converted to NPs using sodium periodate (NaIO_4_). In the presence of NaIO_4_ at pH 7.4, catechol to quinone oxidation induces the formation of covalently cross-linked networks yielding spherical NPs. The yield of D-BSA NPs formation was found to be 65%. Figure 3(A) shows an SEM image of spherical D-BSA NPs with an average particle size around 100 nm. A narrow size distribution in the range of 70–130 nm was obtained from the analysis of the SEM results with ImageJ software (Figure 3(B)).

In PBS buffer, the hydrodynamic size distribution of NPs obtained with DLS measurements ranged between 60 and 250 nm, with a maximum peak at 106 nm (Figure 3(C)). The polydispersity index (PDI) was found to be 0.30 indicating a narrow size distribution. Since DLS measures the hydrodynamic size of NPs instead of dried particle size (SEM) and/or protein NPs can swell with water, DLS results show slightly higher results compared to results obtained from the SEM analysis.^56,57^ The zeta potential of D-BSA NPs in ultrapure water was found to be −28 mV (conductivity: 0.0128 mS/cm) due to the negative surface zeta potential of D-BSA proteins (−7 mV) (Figure 3(D)). A large zeta potential (±30 mV) prevents the particles from aggregating and provides colloidal stability in the suspension.^58^ In the literature, steric stabilization is also obtained when the nanoparticles are covered with large molecules e.g. surfactants and polymers.^59^ Here, we assumed that electrostatic repulsion forces are the main reason for having colloidal stability since the surface of D-BSA NPs is uncovered. In addition, due to the large negative zeta potential of surface (−28 mV), hydration layers around NPs may also help in stabilization.^59^

The structural stabilities of D-BSA NPs in water and PBS buffer at pH 4.0, 7.4, and 9.0 over 4 weeks were analyzed using DLS measurements (Figures S2 and S3). Also, zeta potential measurements in water at different pH values were performed. Figure S2 showed that at pH 7.4 and 9.0 the hydrodynamic sizes and zeta potentials of D-BSA NPs did not change significantly over 4 weeks, indicating the stability of the NPs at pH 7.4 and 9.0. However, at pH 4.0, the hydrodynamic sizes for both measured in water and PBS changed over time. Their PDI values also changed and reached greater values than the PDI values obtained at pH 7.4 or 9.0. This shows the instability of the NPs at pH 4.0. Also, the zeta potential at pH 4.0 could not be measured after 1 week.

The effect of the pH on the NP formation was studied with two approaches; the pH of the medium was varied before or after the addition of the oxidizing agent. The effects of the pH on the BSA structure have been studied several times in the literature. For example, small-angle X-ray scattering data show that BSA maintains its native state (heart shape) from pH 4.0 up to 9.0.^60^ In addition, the circular dichroism (CD) analyses revealed that α-helix content changes slightly from 68% to 66% and 62% when the pH of the medium changed from pH 7.4 to 9.0 and 4.0, respectively.^61^ Although the conformation does not change significantly in this pH range, the addition of high amounts of acetone (66% (v:v)) denatures the protein and causes aggregation.

First, D-BSA proteins were dissolved in water at pH 4.0, 7.4, or 9.0, and then the desolvating agent of acetone and oxidizing agent of NaIO_4_ were added. However, NP formation was not achieved when acidic or basic medium was used at the beginning other than pH 7.4. Larger aggregates were obtained when the starting pH of the medium was adjusted to 4.0. A similar result was obtained when NaIO_4_ was not used as an oxidant. This suggests that at pH 4.0 dopamine-based cross-linking does not occur enough, and only desolvation-induced protein aggregation occurs. On the other hand, after dissolving D-BSA proteins at pH 9.0, the catechol groups were already oxidized to quinone, and a transparent brown color was formed before NaIO_4_ addition. After the addition of NaIO_4_, a clear solution remained, and NPs could not be obtained. Both results obtained at pH 4.0 and 9.0 showed that the pH value of 7.4 is important for NP formation.

**Figure 4:**
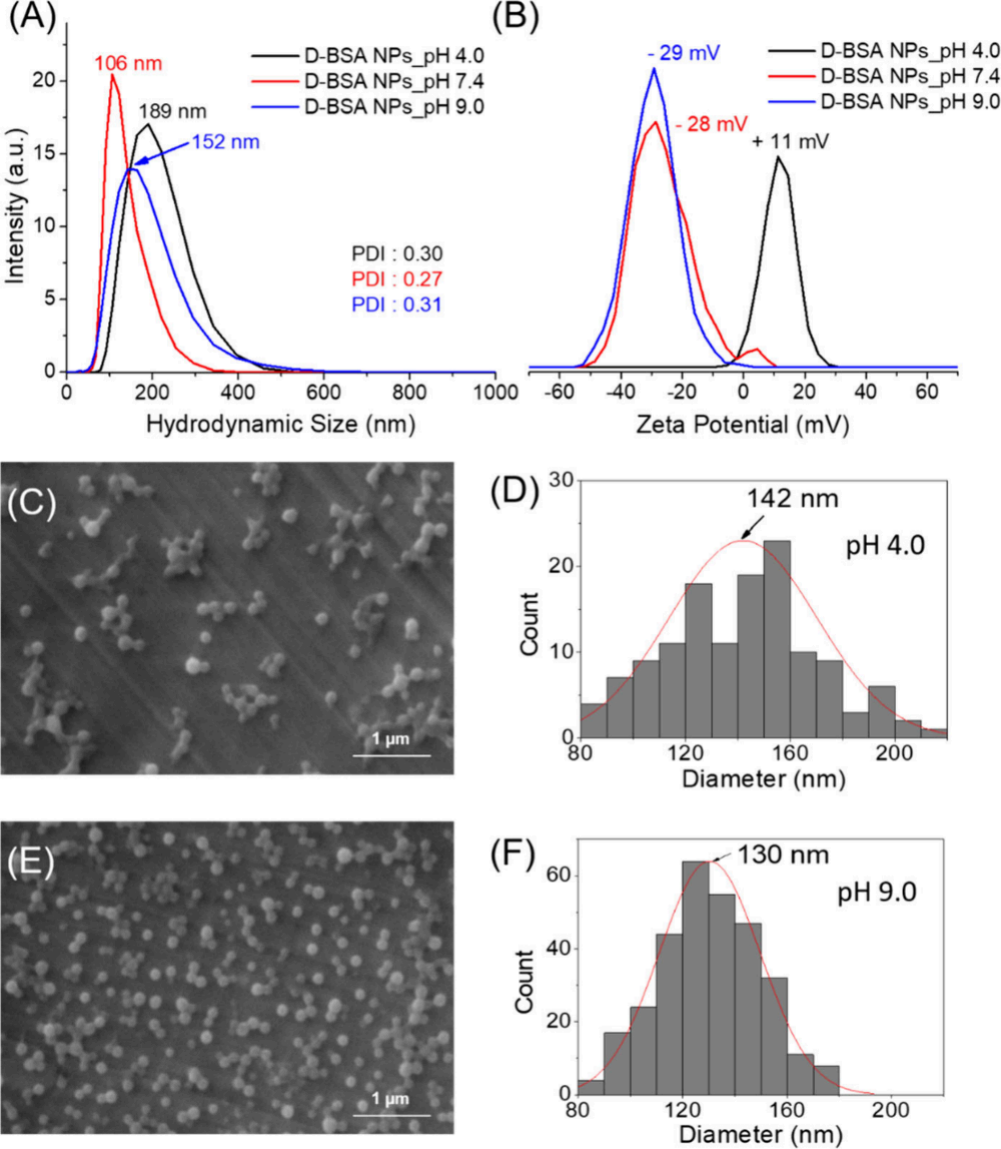
(A) Hydrodynamic size distributions and (B) zeta potentials of D-BSA NPs synthesized at pH 4.0 (black), pH 7.4 (red), and pH 9.0 (blue) in aqueous solutions after addition of NaIO_4_ at pH 7.4. (C) SEM images of D-BSA NPs prepared at pH 4.0 and (D) particle size distribution obtained from the SEM image (C). (E) SEM images of D-BSA NPs prepared at pH 9.0 and (F) particle size distribution obtained from the SEM image (E).

Second, the pH of the medium was kept constant at pH 7.4 or changed from pH 7.4 to 4.0 or 9.0 after the addition of the desolvating agent acetone and the oxidizing agent of NaIO_4_. The pH of medium decreased to pH 4.0 and increased to 9.0 using HCl and NaOH, respectively. Different from the results of the first approach, NPs were obtained when the pH of the medium was changed from 7.4 to 4.0 or 9.0 after the addition of acetone and NaIO_4_. This shows that using an oxidizing agent at pH 7.4 is crucial to obtain NPs. Cationic NPs were obtained when the pH was decreased from 7.4 to 4.0 after adding the oxidizing agent. According to the DLS results, the particle size was found to be around 189 nm which is larger than that obtained at pH 7.4 (106 nm) (Figure 4(A)), and the zeta potential of NPs was found to be +11 mV in ultrapure water (Figure 4(B)). Also, their SEM image revealed a size distribution in the range of 80–220 nm with an average particle size around 146 nm (Figure 4(C) and (D)). UV–vis spectroscopy can be used to analyze the cross-linking of proteins, but the aggregation that occurs after the addition of acetone causes turbidity. Therefore, analysis of D-BSA proteins with NaIO_4_ was performed without acetone (Figure 1(C)). Cencer et al. showed that after the addition of NaIO_4_, catechol was oxidized to quinones, and then quinones converted to quinone methide under acidic pH.^53^ The quinone methide can interact with the nucleophilic sites (−NH_2_ or −SH) of albumins, causing cross-linking of proteins in the acidic environment.

On the other hand, the zeta potential of NPs prepared at pH 9.0 (−29 mV) is similar to the results obtained at pH 7.4 (−28 mV) (Figure 4(B)). The particle sizes were found to be somewhat larger (152 nm (DLS) and 130 nm (SEM)) compared to those obtained at pH 7.4 (106 nm (DLS) and 101 nm (SEM)) (Figures 3 and 4). At pH values of 7.4 or 9.0, quinones can either follow dicatechol formation (at 280 and 485 nm) or α,β-dehydrodopamine formation (at 320 nm) for cross-linking steps. Since extra catechol groups can be auto-oxidized at pH 9.0, the NP formation yield at pH 9.0 is less than that at pH 7.4. The yields of D-BSA NPs formations were found to be 50%, 65%, and 30% at pH 4.0, 7.4, and 9.0, respectively.

In addition to acetone, other organic solvents such as methanol, ethanol, or propanol were used as desolvating agents to obtain D-BSA NPs. However, undissolved aggregates were obtained at the end of desolvation when methanol, ethanol, or propanol was used. Acetone was the only one to yield NPs. The polar aprotic solvent of acetone can form hydrogen bonds with hydrogen donor groups of amino acids of D-BSA proteins, including catechol hydroxyl groups. In addition, a higher dipole moment of acetone (μ = 2.90) compared to dipole moments of others, methanol (μ = 1.70), ethanol (μ = 1.70) and propanol (μ = 1.60), increases the electrostatic interaction between acetone and albumin surface.^62^ These interactions may lead to spherical nano sized particles without forming larger aggregates.

### Biodegradability, Biocompatibility, and Antioxidant Properties of D-BSA NPs

**Figure 5:**
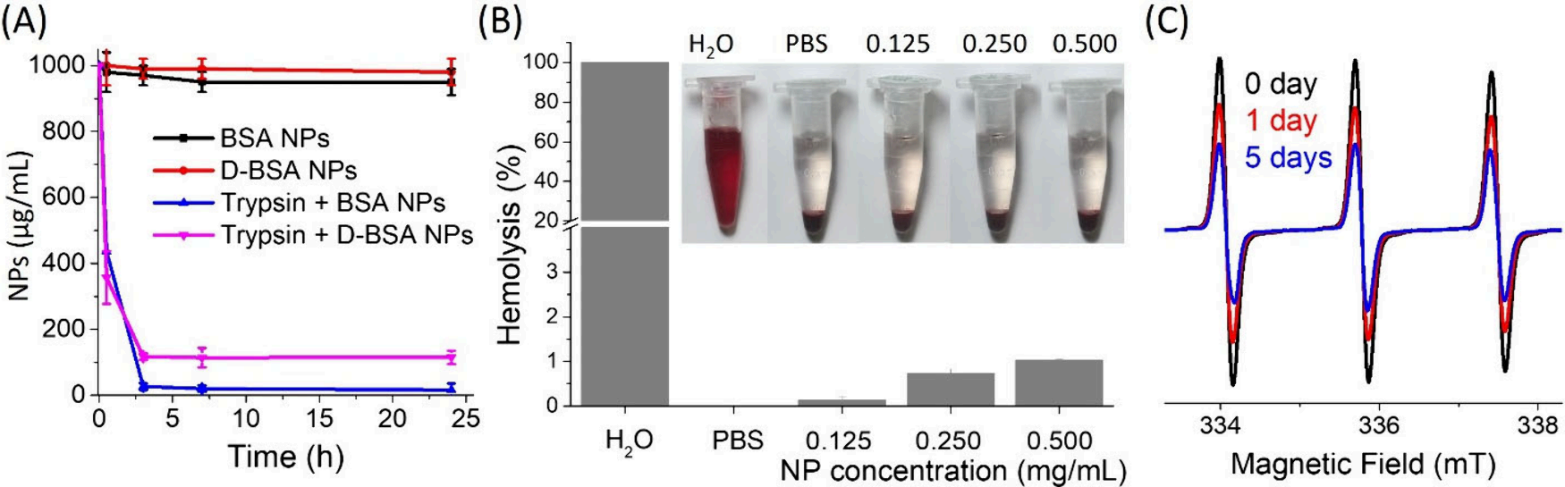
(A) Concentrations of the BSA NPs (black) and D-BSA NPs (red) in the absence of trypsin and BSA NPs (blue) and D-BSA NPs (pink) in the presence of trypsin in water over 24 h. The final concentration of trypsin is 50 μg/mL. (B) Hemolysis percentages of RBCs were plotted after being treated with water (positive control, 100%), PBS (negative control, 0%), 0.125, 0.250 or 0.500 mg/mL D-BSA NPs. Images of the supernatants are displayed at the top of the graph. (C) Time dependent EPR spectra of the TEMPOL radical after addition of D-BSA NPs after 0 days (black), 1 day (red), and 5 days (blue) in 0.01 M PBS at pH 7.4.

The biodegradability of the obtained NPs was studied after digestion with trypsin. UV–vis absorption spectroscopy allows for the monitoring of turbidity of colloidal suspension of NPs.^63,64^ Degradation of NPs reduced turbidity, and thus, decreasing of the signal at 565 nm was observed over time. Although, insignificant degradation of 1000 μg/mL D-BSA NPs was observed in the absence of trypsin, 65% and 90% of D-BSA NPs were degraded after 1 and 3 h, respectively in the presence of 50 μg/mL trypsin (Figure 5(A)). This shows the fast biodegradability of D-BSA NPs at a physiological pH with time. Additionally, the biodegradability pattern of D-BSA NPs was compared to the result of classically prepared BSA NPs using glutaraldehyde. BSA NPs were prepared via the desolvation method with acetone followed by glutaraldehyde cross-linking (Supporting Information).^24^ The particle size and zeta potential results of the BSA NPs and D-BSA NPs are very similar. In PBS buffer, the hydrodynamic size distribution of BSA NPs obtained with DLS measurements ranged between 60 and 300 nm, with a maximum peak at 105 nm. The polydispersity index (PDI) was found to be 0.15, indicating a narrow size distribution. The zeta potential of BSA NPs was found to be −25 mV (Figure S4). Figure 5(A) shows that D-BSA NPs and BSA NPs with similar sizes and zeta potentials have comparable biodegradation patterns despite having different cross-linking mechanisms. Likewise, insignificant degradation of BSA NPs was observed in the absence of trypsin, but 57% and 98% of BSA NPs were degraded after 1 and 3 h, respectively, in the presence of 50 μg/mL trypsin.

To test the biocompatibility of the synthesized NPs, an ex vivo hemolysis test was conducted. Figure 5(B) shows the pictures of obtained supernatants of positive and negative controls, as well as all concentrations tested. When red blood cells (RBCs) obtained from mouse blood were suspended in PBS and incubated with D-BSA NPs at concentrations of 0.500 mg/mL or 0.250 mg/mL for 4 h under shaking conditions, 1% and 0.73% hemolysis results (normalized to hemolysis caused by H_2_O) were observed, respectively. When 0.125 mg/mL NP was used, hemolysis was close to none. The lack of hemolytic activity on mouse RBCs supports the biocompatibility of D-BSA NPs.

Dopamine conjugated albumin NPs showed a radical quenching activity due to the antioxidant feature of the catechol group. A stable nitroxide based TEMPOL radical was mixed with D-BSA NPs with a molar ratio of 1:1 and monitored with EPR spectroscopy over time. Figure 5(C) shows the EPR spectra of TEMPOL before and after the addition of D-BSA NPs. The EPR signal intensity of TEMPOL decreased by 27% and 50% after 1 day and 5 days of mixing with D-BSA NPs, respectively. On the other hand, the signal intensity of TEMPOL decreased by 5% and 11% after 1 day and 5 days of mixing with traditional BSA NPs prepared by using glutaraldehyde as a cross-linker, respectively (Figure S5).^24^ The radical quenching activity of D-BSA NPs originates from the catechol groups on the D-BSA protein. Upon oxidation with IO_4_^–^, covalently formed dicatechol groups and/or unaffected catechol groups provide an antioxidant feature to the NPs. A weak but detectable signal decrease observed with BSA NPs could be originated from the cysteine amino acids of albumin protein. In the literature, albumin NPs are frequently employed to deliver antioxidants into the body through the encapsulation of these molecules.^65−67^ The administration of antioxidants is crucial to reduce the risk of diseases, including several types of cancer and cardiovascular conditions, by reducing the cell damage caused by free radicals. From this aspect, D-BSA NPs possess the capability to scavenge free radicals and provide antioxidant properties in addition to the drug delivery function without loading external antioxidant molecules. To our knowledge, no studies have shown the antioxidant effect of dopamine within albumin particles. However, the antioxidant properties of dopamine have been reported in different systems such as after it has been conjugated with hydrogels or incorporated into lipid systems.^68,69^

### Drug Binding and Release Properties of D-BSA NPs

**Figure 6:**
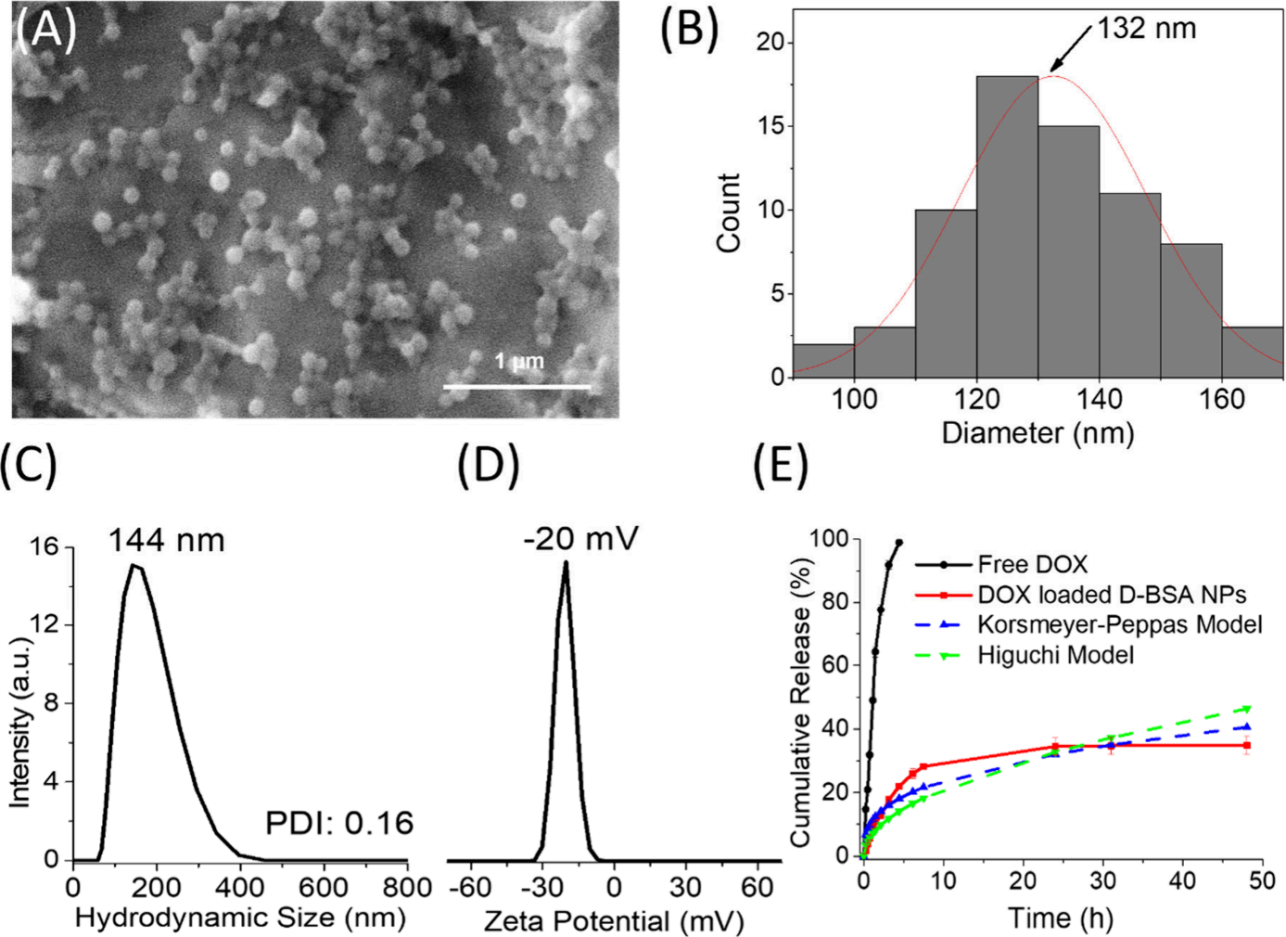
(A) SEM image of DOX loaded D-BSA NPs (pH 7.4) and (B) particle size distribution obtained from the SEM image (A). (C) DLS results of the hydrodynamic size distribution of DOX loaded D-BSA NPs in PBS at pH 7.4. (D) Zeta potential of DOX loaded D-BSA NPs in aqueous solutions at pH 7.4. (E) Cumulative amount of free DOX (black) and released DOX from D-BSA NPs (red) in 0.01 M PBS buffer at 37 °C, pH 7.4, diffused from the D-tube dialyzer. Data obtained from the released DOX were fitted with Korsmeyer-Peppas (blue dashed line) and Higuchi (green dashed line) models.

Doxorubicin (DOX), which is an anticancer chemotherapy drug, was used to study the drug binding and release properties of D-BSA NPs. DOX loading was achieved via an incubation method after NP formation. DOX loading was applied to the NPs prepared at pH 7.4 medium due to the results of a smaller size and higher formation yield compared to those obtained with other pH mediums, e.g., 4.0 and 9.0. DOX entrapment efficiency and loading capacity of D-BSA NPs for a 15:1 DOX/D-BSA protein molar ratio were 87% ± 1.7 and 9.1% ± 0.2, respectively. The size of D-BSA NPs did not change significantly upon DOX loading (Figure 6(A-C)). According to SEM results, the average size of D-BSA NPs increased from 101 to 132 nm, and according to DLS results, the maximum intensity of the hydrodynamic size increased from 106 to 144 nm. After DOX loading, the zeta potential of D-BSA NPs changed from −28 to −20 mV in ultrapure water. The conductivity of D-BSA NPs and DOX loaded D-BSA NPs solutions was found to be similar, 0.0128 and 0.0108 mS/cm, respectively (Figure 6(D)). Since the conductivity values are so similar, the change in the zeta potential could be due to the changes in the surface of NPs e.g., coating with drugs.

The release rate of DOX from D-BSA NPs was investigated by using a D-tube dialyzer. The result was compared with the diffusion rate of free DOX from the dialyzer tube to evaluate the impact of NPs on drug release (Figure 6(E)). While free DOX exhibited complete release within 5 h, DOX release from D-BSA NPs demonstrated 34% release at the end of 24 h and remained constant thereafter. These results indicate that DOX is released in limited amounts via diffusion when it is loaded onto D-BSA NPs. In addition, the DOX release mechanism of D-BSA NPs was investigated using two mathematical models: the Korsmeyer-Peppas model based on cumulative % drug release vs n power of time and the Higuchi model based on cumulative % drug release vs square root of time.^70^ The plots obtained by fitting the release data with Korsmeyer-Peppas and Higuchi models are shown in Figure 6(E). The correlation value (R^2^) obtained from the Korsmeyer-Peppas model is larger than the value obtained from the Higuchi models (0.95 vs 0.92). Also, the release power (n) value was found to be 0.34 from the Korsmeyer-Peppas model. The n values less than 0.43 indicate the presence of the Fickian diffusion mechanism.^71^ On the other hand, drug release dynamics from NPs in PBS buffer should be different in cell culture media due to the presence of enzymes, proteins, lipids, vitamins, etc. which can change the release profile through interactions with the nanoparticle or bound drug.

### In Vitro Efficacy of NPs

**Figure 7:**
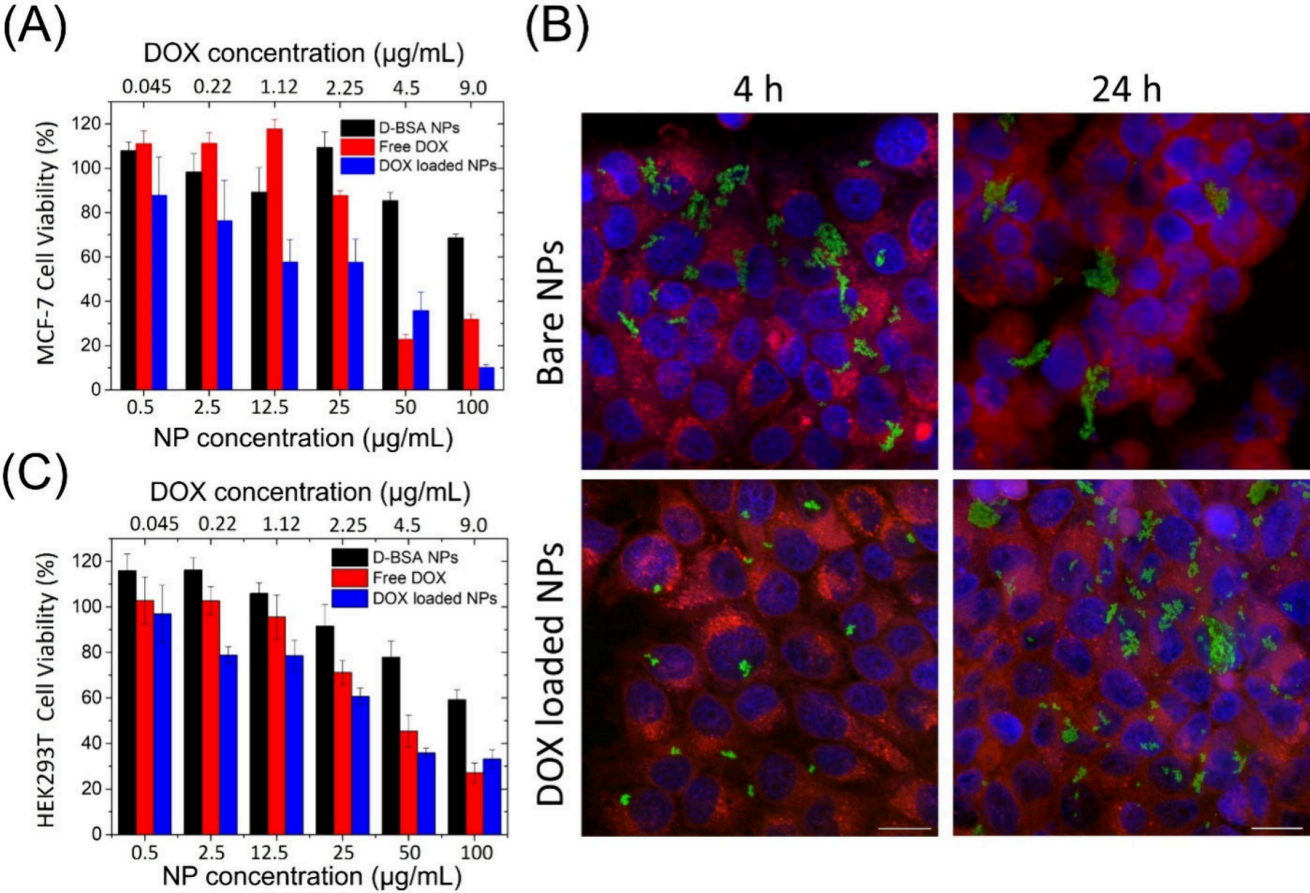
(A) MCF-7 cell viability upon addition of D-BSA NPs (black), DOX loaded BSA NPs (blue), or free DOX (red) at different concentrations after 24 h of incubation. The increase in MCF-7 cell viability observed when the D-BSA NPs concentration increased from 12.5 μg/mL to 25 μg/mL was found to be insignificant, *p* > 0.05. (B) MCF-7 cellular uptake of 25 μg/mL bare (top) and DOX loaded (bottom) FITC labeled D-BSA NPs for 4 h (left) and 24 h (right) of treatments. MCF-7 cells were stained with Dil (red) vital dye. At the end of the treatment, cells were stained with DAPI (blue) to highlight the nucleus. Scale bar = 20 μm. (C) HEK293T cell viability upon addition of D-BSA NPs (black), DOX loaded BSA NPs (blue), or free DOX (red) at different concentrations after 24 h of incubation.

The effect of D-BSA NPs, DOX loaded D-BSA NPs, and free DOX on survival of MCF-7 breast cancer cells was investigated after 24 h of treatment (Figure 7(A)). At low concentrations, D-BSA NPs did not reduce the viable cell number significantly, but at higher concentrations of D-BSA NPs e.g. 50 μg/mL and 100 μg/mL, the cell viability reduced to 85% and 70%, respectively. It was observed that while 25 μg/mL unloaded D-BSA NPs did not significantly change the cell viability, DOX loaded D-BSA NPs at the same NP concentration (containing 2.25 μg/mL of DOX) reduced the cell viability to 50%. Cellular uptake of DOX loaded FITC labeled D-BSA NPs and unloaded NPs was investigated in MCF-7, after incubation with 25 μg/mL NPs for 4 and 24 h (Figure 7(B)). For both unloaded and drug loaded NPs, cellular uptakes were observed after 4 h, and more entrances were observed after 24 h. Also, DOX loaded NPs displayed well dispersed cells after 24 h. The resulting particle sizes around 100–140 nm are suitable for the endocytosis of NPs.^72^ It has been shown that endocytosis of HSA NPs (<200 nm) to human retinal pigment epithelial (ARPE-19) cells was facilitated by mainly caveolae and clathrin pathways.^73^

Remarkably, it was observed that the cell viability of MCF-7 decreased gradually when treated with 0.5–100 μg/mL DOX loaded D-BSA NPs. The addition of 100 μg/mL D-BSA NPs containing 9.0 μg/mL DOX decreased the cell viability to 10%. The effectiveness of DOX carried by D-BSA NPs was also compared with free DOX in the range of 0.045 to 9.0 μg/mL, the same amounts of DOX carried by NPs. The addition of free DOX to MCF-7 cells in the range of 0.045–1.12 μg/mL had no strong influence on the cell viability after 24 h of treatment. A comparison of in vitro efficacy of free DOX and DOX loaded NPs on MCF-7 cells showed that at low concentrations e.g. 12.5 μg/mL NPs (including 1.12 μg/mL DOX), the viability reduced to 57% when DOX loaded NPs were used, but treatment with free DOX did not reduce the viability. This suggests efficient cellular uptake of albumin NPs compared with free drug molecules. After 48 h of treatment, again the efficacy of DOX loaded NPs is better than the free DOX at low concentrations, but it is similar at higher concentrations (Figure S7). Nevertheless, the cell viability decreased sharply to around 20–30% upon addition of maximum amounts of free DOX (9.0 μg/mL) used in this study. As a result, higher killing results with a concentration dependence were obtained when DOX was delivered with D-BSA NPs, indicating that D-BSA NPs are a good candidate for drug delivery.

Additionally, after 1 month of storage at 4 °C as a powder, D-BSA NPs and DOX loaded D-BSA NPs were applied to MCF-7 cells for 24 h to check their stability. Still, DOX-loaded NPs gradually decrease in viability with concentration indicating their stability during at least one month of storage (Figure S8).

The cytotoxicity of the D-BSA NPs was also studied with noncancerous human embryonic kidney cell line HEK293T (Figure 7(C)). After incubation of HEK293T cells with different amounts of D-BSA NPs for 24 h, the cell viability did not change significantly at low concentrations (<25 μg/mL) but decreased to 60% when 100 μg/mL D-BSA NPs were used. On the other hand, addition of DOX loaded D-BSA NPs at different concentrations induced a gradual cell death, and a similar trend was observed when MCF-7 cells were used. After addition of 100 μg/mL DOX loaded D-BSA NPs (consisting of 9 μg/mL DOX), the HEK293T cell viability decreased to 33%. Furthermore, the same amount of free DOX caused HEK293T cell death very similar to that caused by DOX loaded D-BSA NPs (Figure 7(C)).

## Conclusions

Dopamine (D) conjugation provides an alternative method to glutaraldehyde-mediated cross-linking in the preparation of albumin NPs. The use of glutaraldehyde has disadvantages, such as toxicity and the possibility of undesirable interactions between glutaraldehyde and drugs. Here, first BSA proteins were conjugated with dopamines, and then the desolvation method was applied. To obtain stable NPs, D-BSA proteins were covalently cross-linked via the catechol sides of dopamines upon addition of an oxidizing agent of NaIO_4_. After optimization of the experimental parameters including the pH (7.4), dopamine:IO_4_^–^ ratio (2:1), desolvating agent (acetone), and acetone:water volume ratio (4:1), we obtained spherical D-BSA NPs around 100 nm in size with uniform size distribution. Dicatechol formation detected from UV–vis measurements of D-BSA proteins at pH 7.4 should lead to the NP stabilization. The formation yield was found to be 65%. The high surface zeta potential of NPs (−28 mV) provides for obtaining a stable colloidal suspension within at least one month.

Moreover, the obtained NPs show antioxidant features due to the catechol groups of dopamines on the NPs. EPR measurements showed that the TEMPOL radical signal intensity decreased to 50% upon addition of D-BSA NPs at a 1:1 molar ratio after 5 days. However, BSA NPs obtained by using glutaraldehyde decreased the radical signal intensity by only 11%. On the other hand, biodegradability of D-BSA NPs and classical BSA NPs was found to be similar. A fast degradation was observed after 3 h for both NPs having different cross-linking mechanisms. Drug loading and release abilities of D-BSA NPs were also studied with doxorubicin (DOX). High DOX loading capacity (9.1%) and a limited diffusion release (34%) after 48 h indicate that D-BSA NPs could be used as drug carriers. The effectiveness of DOX loaded D-BSA NPs on cell death was investigated with MCF-7 breast cancer cells. At low concentrations of DOX (up to 2.25 μg/mL), cell death was observed (reached 50%) when the drug was delivered together with D-BSA NPs, but cell death was not observed with free DOX in this range. Also, at high concentrations such as 100 μg/mL D-BSA NPs containing 9.0 μg/mL DOX, the cell viability decreased to 10% when they were incubated with cells for 24 h. In conclusion, having an antioxidant feature, biodegradability, biocompatibility, high doxorubicin (DOX) loading, limited drug release, and a pronounced effect on the viability of MCF-7 breast cancer cells after DOX loading suggests that D-BSA NPs prepared by catechol cross-linking could be used in drug delivery studies.

## Supporting Information Available

Additional experimental details, characterization of D-BSA protein using MALDI-TOF mass spectrometry, ATR-FTIR spectroscopy, and zeta potential measurements, stability tests over 4 weeks, characterization of BSA NPs, time dependent EPR spectra of TEMPOL radical after addition of traditional BSA NPs, calibration curves of NPs obtained from UV–vis absorption spectroscopy measurements at different concentrations and cell viability results (PDF)
