# Supplementary material for: Periodate-Mediated Cross-Linking for the Preparation of Catechol Conjugated Albumin Nanoparticles Used for in Vitro Drug Delivery
Source: ACS Appl Bio Mater. 2025 Feb 14;8(3):2182–93. doi: 10.1021/acsabm.4c01737 (PMC11931531; doi:10.1021/acsabm.4c01737)
Supplement: Supplementary file 1 — mt4c01737_si_001.pdf [file mt4c01737_si_001.pdf]

SUPPORTING INFORMATION

Periodate-mediated crosslinking for the preparation of catechol conjugated albumin nanoparticles used for in vitro drug delivery

Eda Argitekin[†], Ozlem Erez[‡], Gulcin Cakan-Akdogan[‡,§] and Yasar Akdogan[†,]*

[†]Materials Science and Engineering Department, Izmir Institute of Technology, Izmir, Turkiye

[‡]Izmir Biomedicine and Genome Center, Izmir, Turkiye

[§]Department of Medical Biology, Faculty of Medicine, Dokuz Eylul University, Izmir, Turkiye

*Corresponding Author

Email: yasarakdogan@iyte.edu.tr

Preparation of BSA NPs

62.5 mg BSA was dissolved in 1 mL deionized water. Afterwards, acetone was added dropwise to BSA solution using a syringe pump with a flow rate of 1.0 mL/min. The acetone:water ratio was adjusted to 1:4 (v/v). Then, 18 µL of 8% (v/v) cross-linking agent glutaraldehyde was added and the solution was stirred overnight at 750 rpm. Aliquots NP solutions were precipitated by centrifugation at 14,000 rpm for 45 min.

Pellets were washed with ultra-pure water to eliminate acetone, unreacted BSA and glutaraldehyde.

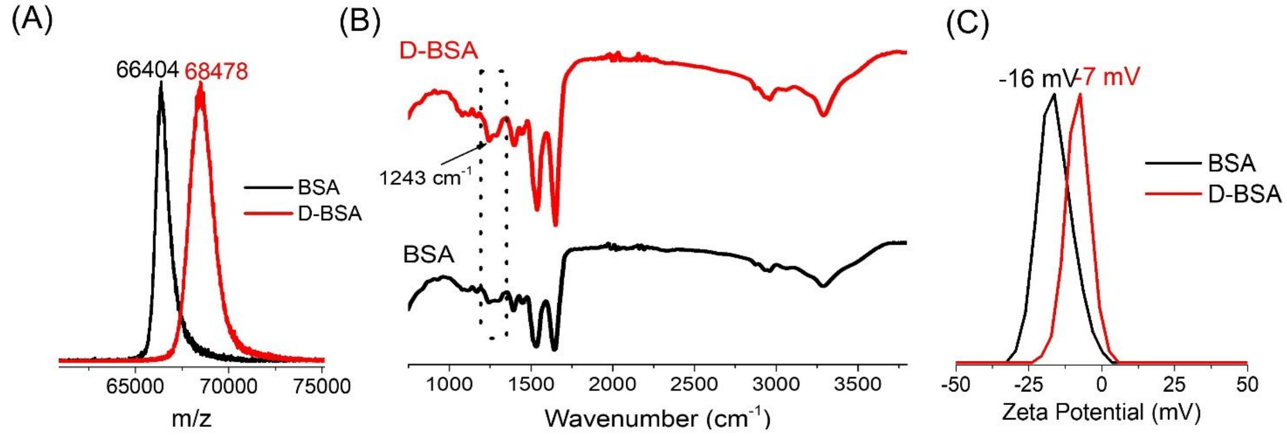

Figure S1. (A) MALDI-TOF mass spectra of BSA (black) and dopamine-conjugated BSA (D-BSA) (red) proteins. (B) ATR-FTIR spectra of BSA (black) and D-BSA (red). (C) Zeta potentials of BSA (black) and D-BSA (red) in water.

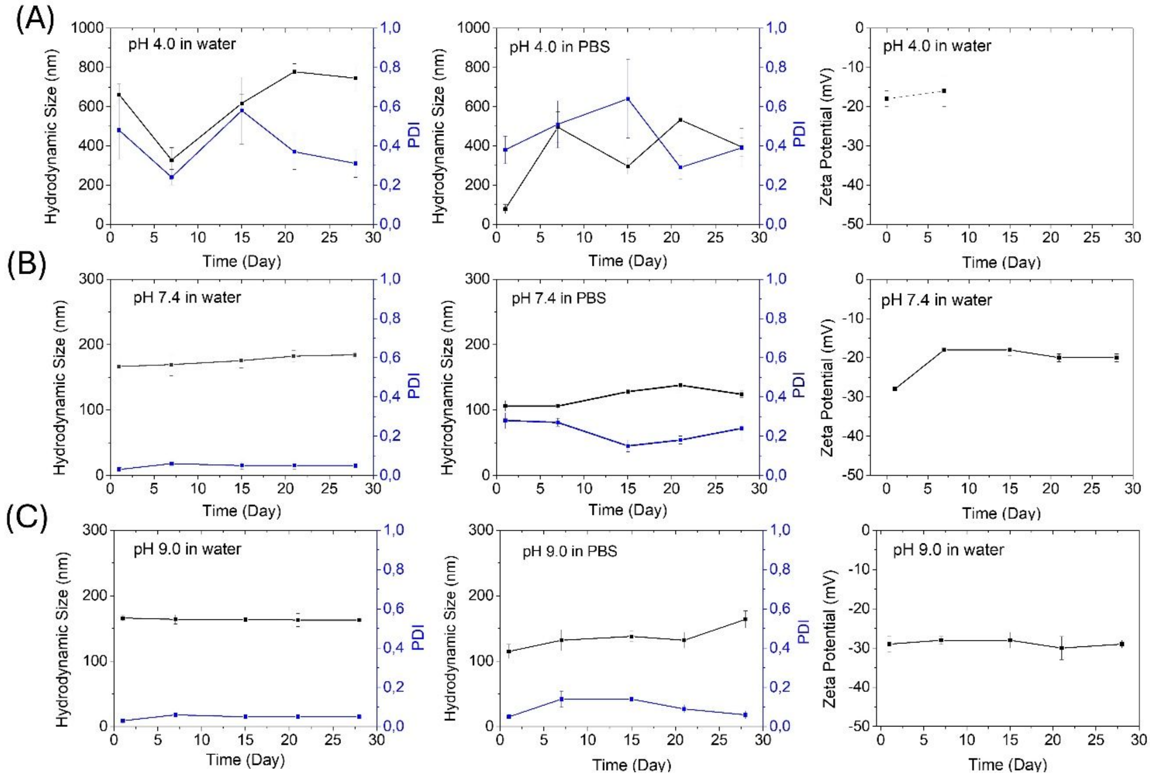

Figure S2. DLS results of hydrodynamic sizes in water and PBS, and zeta potentials of D-BSA NPs over four weeks at pH 4.0 (A), pH 7.4 (B) and pH 9.0 (C).

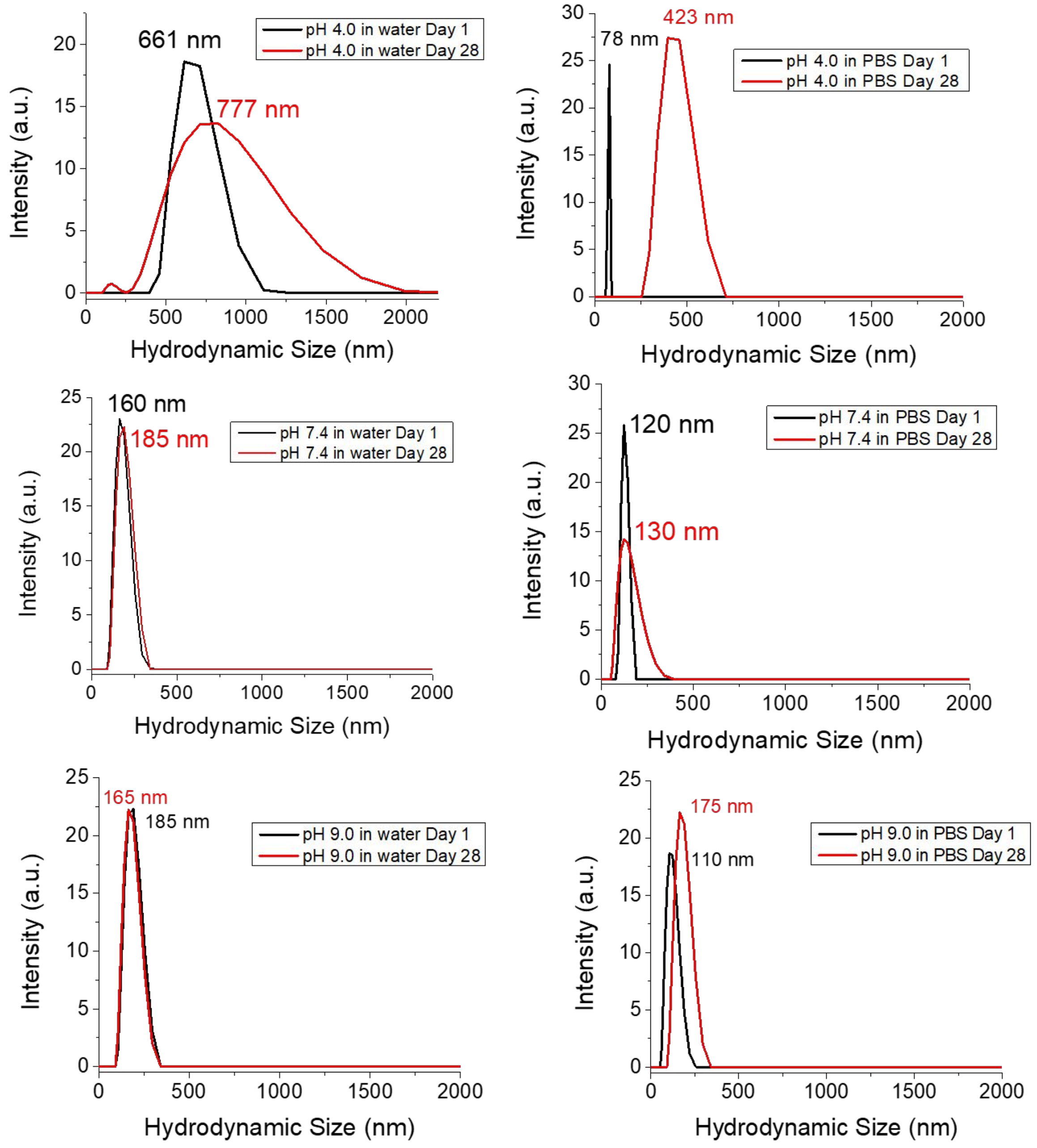

Figure S3. DLS results of hydrodynamic size distributions of D-BSA NPs after 1 day and 28 days at pH 4.0, pH 7.4 and pH 9.0 in water (left) and PBS (right).

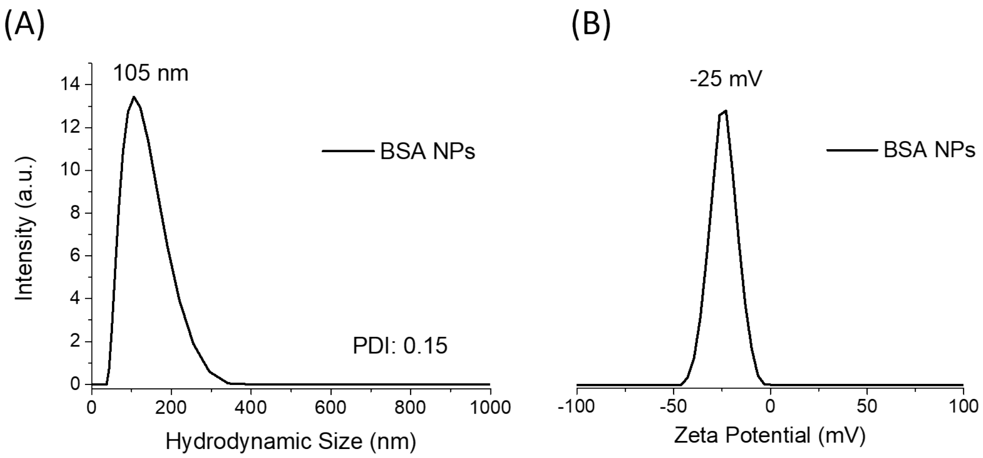

Figure S4. DLS results of hydrodynamic size distribution (A) and zeta potential (B) of BSA NPs synthesized by desolvation method followed by glutaraldehyde for crosslinking.

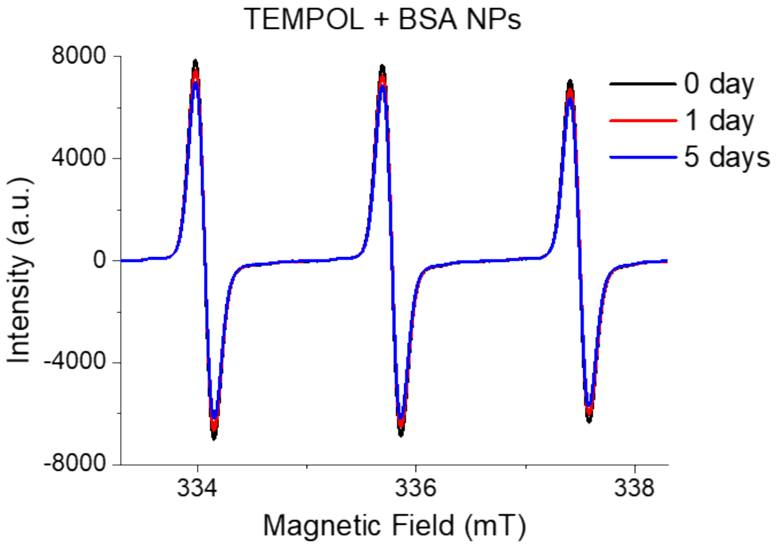

Figure S5. Time dependent EPR spectra of TEMPOL radical after addition of traditional BSA NPs after 0 day (black), 1 day (red) and 5 days (blue) in 0.01 M PBS at pH 7.4.

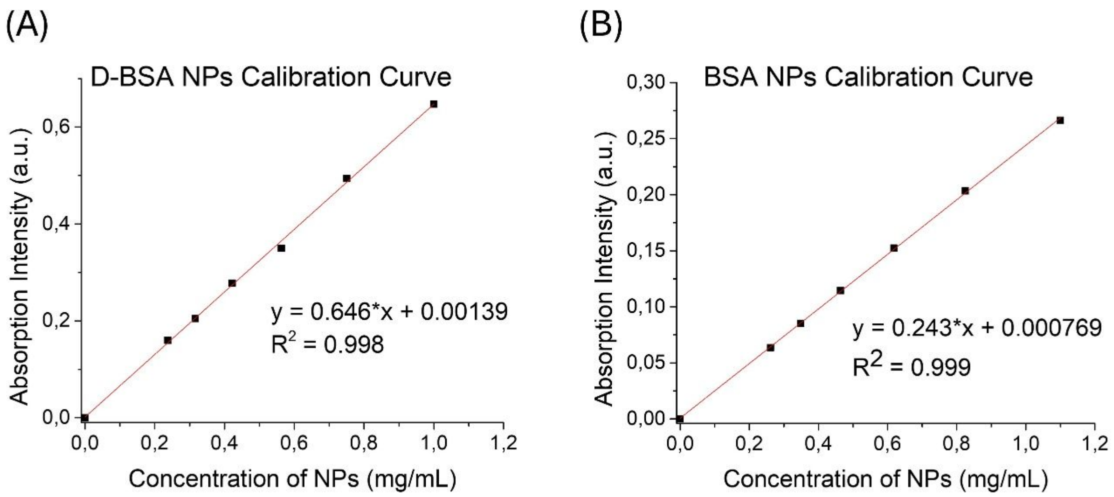

Figure S6. Calibration curves of D-BSA NPs (A) and BSA NPs (B) obtained from UV-Vis absorption spectroscopy measurements at different concentrations. Absorption intensities increase versus NPs concentration due to scattering light effect of nanoparticles in the colloidal solution.

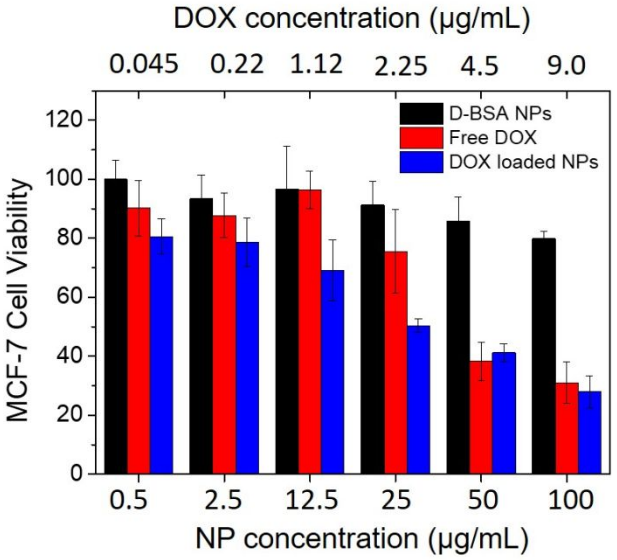

Figure S7. MCF-7 cell viabilities upon addition of D-BSA NPs (black), DOX loaded BSA NPs (blue) or free DOX (red) at different concentrations after 48 hours incubation.

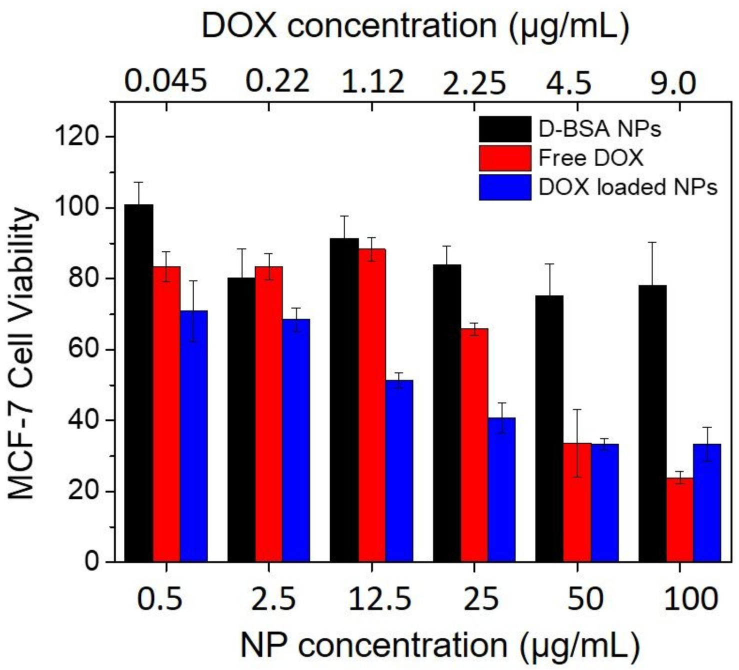

Figure S8. MCF-7 cell viabilities upon addition of 4 weeks aged D-BSA NPs (black), DOX loaded BSA NPs (blue) or free DOX (red) at different concentrations after 24 hours incubation.
